# Myeloid-Derived Suppressor Cells and γδT17 Cells Contribute to the Development of Gastric MALT Lymphoma in *H. felis*-Infected Mice

**DOI:** 10.3389/fimmu.2019.03104

**Published:** 2020-01-28

**Authors:** Yanan Zhao, Fei Lu, Jingjing Ye, Min Ji, Yihua Pang, Yan Wang, Lingbo Wang, Guosheng Li, Tao Sun, Jingxin Li, Daoxin Ma, Chunyan Ji

**Affiliations:** ^1^Department of Hematology, Qilu Hospital of Shandong University, Jinan, China; ^2^Department of Hematology, Taian Central Hospital, Taian, China; ^3^Department of Geriatric Medicine, Qilu Hospital of Shandong University, Jinan, China; ^4^Department of Physiology, Medicine School of Shandong University, Jinan, China

**Keywords:** MDSCs, γδT17, immunosuppression, MALT lymphoma, murine model

## Abstract

*Helicobacter*-induced chronic inflammation and immune disorders are closely associated with the development of gastric mucosa-associated lymphoid tissue (MALT) lymphoma. Myeloid-derived suppressor cells (MDSCs) exhibit strong immunosuppressive properties and promote the growth of various solid tumors. However, the role of MDSCs in the development of MALT lymphoma has not been elucidated so far. We detected significant infiltration and enrichment of MDSCs in patients with MALT lymphoma, as well in *Helicobacter felis*-infected mouse model of gastric MALT lymphoma. In addition, the expression of arginase-1 and inducible nitric oxide synthase was significantly elevated both in gastric MALT lymphoma tissues and *H. felis*-infected stomach. Persistent *H. felis* infection closely reproduced the development of gastric MALT lymphoma and was accompanied by increased numbers of γδT17 cells. Accumulation of γδT17 cells was also validated in the human gastric MALT lymphoma tissues. Furthermore, the elevated cytokines interleukin-23 and interleukin-1β, as well as chemokines CCL20/CCR6, may be involved in the accumulation of γδT17 cells and the subsequent immunosuppression. These findings highlight the role of MDSCs and γδT17 cells in immune dysregulation during gastric MALT lymphoma development and their potential as therapeutic targets.

## Introduction

Extranodal marginal zone B-cell lymphoma of the mucosa-associated lymphoid tissue (MALT) accounts for 7–8% of all newly diagnosed lymphomas, of which gastric MALT lymphoma is the most common (1). Furthermore, 90% of the gastric MALT lymphoma cases are associated with *Helicobacter pylori* infection (2–4). Since, gastric MALT lymphoma is a typical model of chronic inflammation-induced gastric tumor development (5), it is essential to dissect its underlying immune mechanisms.

Myeloid-derived suppressor cells (MDSCs) are a heterogeneous population of immature cells derived from myeloid progenitors with strong immunosuppressive functions (6). Human MDSCs are commonly defined as CD11b^+^CD33^+^ and lack mature myeloid and lymphoid markers, as well as major histocompatibility complex class II molecule human leukocyte antigen DR isotype (7, 8). Murine MDSCs are characterized by the myeloid-cell lineage differentiation antigens Gr-1 and CD11b (6, 9). MDSCs suppress immune responses in pathological conditions by upregulating arginase 1 (Arg-1), inducible nitric oxide synthase (iNOS) and regulatory T cells (Tregs) (10, 11). Studies show that MDSCs accumulate during inflammation, infection, and in tumors such as multiple myeloma, chronic lymphocytic leukemia, and lymphoma (10, 12). However, the function of MDSCs in MALT lymphoma development remains to be elucidated.

The γδT cells are abundant in the gastrointestinal mucosa, wherein they maintain immune homeostasis. In addition, these cells are activated during *H. pylori-*associated chronic inflammation and drive the immune imbalance in gastric mucosa (13). The γδT17 cell subset in particular modulates immune response in the colorectal cancer and hepatocellular carcinoma microenvironment by recruiting and activating MDSCs (14, 15). Furthermore, the MDSCs may promote γδT17 polarization by secreting IL-23 and IL-1β, which further mediate tumor immune tolerance (15). Therefore, we hypothesized that γδT17 and MDSCs are involved in the development of gastric MALT lymphoma. To this end, we established a mouse model of chronic *Helicobacter*-induced lymphomagenesis and detected MDSCs and γδT17 accumulation in the murine stomach tissues as well as in human MALT lymphoma tissues. Our findings provide new insights into the pathogenesis of gastric MALT lymphoma and identify novel therapeutic targets.

## Materials and Methods

### Patients and Controls

Eight newly diagnosed MALT lymphoma patients (two male and six female; median age, 57 years; age range, 27–76 years) presenting at the Qilu Hospital, Shandong University from May 2017 to March 2018 were enrolled in this study. In addition, 12 healthy donors (6 male and female each; median age, 51 years; age range, 30–68 years) were enrolled as the control group. The study was approved by the Medical Ethical Committee of Qilu Hospital, Shandong University. Detailed clinical information of the patients is summarized in Table 1.

**Table 1:** Clinical characteristics of patients and healthy donors.

**Clinical characteristics**	**MALT lymphoma**	**Healthy donor**
Median age (range), years	57 (27–76)	51 (30–68)
Male, *n*	2	6
Female, *n*	6	6
IPI, *n*		
0–1	5	Null
2–3	2	Null
4–5	1	Null
Ann Arbor stage		
I	3	Null
II	1	Null
III	1	Null
IV	3	Null

### *H. felis* Culture and Infection

*H. felis* (American Type Culture Collection 49179) was obtained from the American Type Culture Collection (Manassas, VA, USA), and cultured on trypticase soy agar containing 5% defibrinated sheep blood in a microaerophilic atmosphere for 48 h at 37°C. The bacterial colonies were harvested and resuspended in phosphate-buffered saline (PBS), and analyzed by Gram staining. In addition, bacterial DNA was extracted using TIANamp Bacteria DNA Kit (TIANGEN, China) and sequenced. The density of the bacterial suspension was adjusted to 10^9^ CFU/ml for infecting animals.

### *H. felis* Infection Model

Female 6- to 8-week-old BALB/c mice were purchased from Nanjing Biomedical Research Institute of Nanjing University, and housed in specific pathogen-free animal care facility and closely monitored. The animal experiments were reviewed and approved by the Medical Ethical Committee of Qilu Hospital, Shandong University. The mice were divided into the *H. felis*-infected and control groups and accordingly inoculated with 100 μl of the bacterial suspension (10^8^ CFU) or PBS three times every other day via the intragastric route, as previously described (16). The animals were killed 8, 11, 14, and 19 months after infection.

### Urease Test

Bacterial colonization was assessed from 4 weeks after *H. felis* inoculation. Stomach tissues were excised, cut along the greater curvature, rinsed with saline, and cut into small pieces. Rapid urease test was conducted using a commercial kit (Begen, China) according to the manufacturer's instructions. Appearance of a red color indicated positive result.

### PCR

Bacterial DNA was extracted from the gastric tissues or stool samples using QIAamp cador Pathogen mini kit (Cat. no. 54104) or QIAamp DNA Stool Mini Kit (Cat. no. 51505) as appropriate. The *H. felis FlaB* gene was amplified as previously described (17) using the following primers: FlaB, 5′-TTCGATTGGTCCTACAGGCTCAGA-3′ and 5′-TTCTTGTTGATGACATTGACCAACGCA-3′; glyceraldehyde 3-phosphate dehydrogenase, 5′-GCTAAGCAGTTGGTGGTGCA-3′ and 5′-TCACCACCATGGAGAAGGC-3′.

### Histology and Immunohistochemistry

Longitudinal stomach tissue strips from *H. felis*-infected or uninfected mice were fixed with 10% formaldehyde, embedded in paraffin, and cut into 4-μm-thick sections. Hematoxylin and eosin staining was performed as per standard protocols, and the presence of lymphoid follicles and lymphoepithelial lesions (LELs) were recorded. Sections of murine stomach or human MALT lymphoma tissues were subjected to heat-induced epitope retrieval and then incubated overnight with anti-TCRγδ (Abcam, Cat. no. ab118864), anti-IL-17 (Abcam, Cat. no. ab79056), anti-IL-1β (Abcam, Cat. no. ab9722), anti-IL-23 (Santa Cruz, Cat. no. sc-50303), anti-Arg-1 (Proteintech, Cat. no. 16001-1-AP), anti-iNOS (Proteintech, Cat. no. 18985-1-AP), anti-Gr-1 (R&D systems, Cat. no. MAB1037-100), anti-CD11b (Abcam, Cat. no. ab133357), anti-CD33 (Abcam, Cat. no. ab11032), and anti-CD11b (Abcam, Cat. no. ab133357) primary antibodies at 4°C. The following day, the sections were probed with biotin–streptavidin horseradish peroxidase-conjugated secondary antibody, and stained using 3,3′-diaminobenzidine reagent. For immunofluorescence, fluorochrome-conjugated secondary antibodies were used. Positively stained cells were counted in five random non-overlapping fields (400× magnification; Nikon, Ni-U) using ImageJ software. The histoscore of each biomarker was evaluated according to Table 2.

**Table 2:** Criteria for immunohistochemistry (IHC) score.

**Percentage of positive cells (a)**		**Staining intensity grading (b)**	
**Percentage (%)**	**Score**	**Intensity**	**Score**
<5	0	None	0
6–25	1	Light brown yellow	1
26–50	2	Brown yellow	2
51–75	3	Dark brown yellow	3
More than 76	4		

*Histoscore = a + b*.

### Flow Cytometry

Peripheral blood samples were treated with red blood cell lysis buffer (eBioscience, USA), and the spleens were gently crushed and filtered through a 40-μm nylon mesh strainer to obtain single-cell suspension. The cells were washed and resuspended in PBS and stained with Brilliant Violet 421 anti-mouse Ly-6G/Ly-6C (Gr-1) (Biolegend, Cat. no. 108434), APC/Cy7 antihuman CD45 (Biolegend, Cat. no. 368516), APC antihuman Lineage Cocktail (CD3, CD19, CD20, CD56; Biolegend, Cat. no. 363601), PE antihuman CD33 (Biolegend, Cat. no. 303404), PE-CF594 mouse antihuman leukocyte antigen DR isotype (BD Horizon, 562304), and Brilliant Violet 711 antimouse/human CD11b (Biolegend, Cat. no. 101242) antibodies as appropriate.

For intracellular cytokine staining, the cells were stimulated with phorbol myristate acetate (50 ng/ml) and ionomycin (1 μg/ml) in the presence of monensin (2 μM, Invitrogen ThermoFisher Scientific) and Brefeldin A (3 μg/ml, Invitrogen ThermoFisher Scientific) at 37°C for 4 h. After staining with APC/Cy7 antimouse CD45 (Biolegend, Cat. no. 103116), APC/Cy7 antihuman CD45 (Biolegend, Cat. no. 304014), PE/Cy7 antimouse CD3 (Biolegend, Cat. no. 100220), PE/Cy7 antihuman CD3 (Biolegend, Cat. no. 300420), fluorescein isothiocyanate antimouse CD4 (Biolegend, Cat. no. 100406), fluorescein isothiocyanate antihuman CD4 (Biolegend, Cat. no. 300506), PE-CF594 rat antimouse CD8a (BD Horizon, Cat. no. 562283), PE-CF594 mouse antihuman CD8 (BD Horizon, Cat. no. 562282), PE antimouse TCRγ/δ (Biolegend, Cat. no. 118108), and PE antihuman TCRγ/δ (Biolegend, Cat. no. 331210) antibodies, the cells were fixed, permeabilized, and stained with APC anti-IL-17A (eBio17B7) (eBioscience, Cat. no. 17-7177-81) and APC anti-human IL-17A (Biolegend, Cat. no. 512334) antibodies. For staining the Treg marker, the cells were permeabilized as per the manufacturer's instructions (eBioscience, San Diego, CA, USA) before incubating with the anti-Foxp3 antibody. Data were acquired using BD FACSAria III flow cytometer (BD Biosciences, USA) and analyzed with the FlowJo V10 software (Tree Star).

### Real-Time Quantitative Polymerase Chain Reaction

Total RNA from the stomach tissues and spleens were isolated using TRIzol reagent (Invitrogen, Carlsbad, CA, USA) and reverse transcribed using Prime script RT reagent kit (Takara Bio Inc., Japan). Real-time quantitative PCR was performed using 5 μl 2× SYBR Green Real-Time PCR Master Mix (Toyobo, Osaka, Japan), 1 μl complementary DNA, 0.8 μl forward and reverse primers, and 3.2 μl ddH_2_O in a LightCycler 480II PCR machine (Roche, Switzerland) according to the manufacturer's instruction. The PCR conditions were as follows: initial denaturation at 95°C for 3 min, followed by 40 cycles of 95°C for 30 s, 60°C for 30 s, and 72°C for 30 s. The primer sequences are listed in Table 3.

**Table 3:** Primer sets and genes included in real-time quantitative PCR analysis.

**Name**	**Forward primer (5^**′**^-3^**′**^)**	**Reverse primer (5^**′**^-3^**′**^)**
NF-κB (mus)	ATGTAGTTGCCACGCACAGA	CCTGAGCCATAGAGTGCAGC
IL-17 (mus)	TTTAACTCCCTTGGCGCAAAA	CTTTCCCTCCGCATTGACAC
IL-23 (mus)	ATGCTGGATTGCAGAGCAGTA	ACGGGGCACATTATTTTTAGTCT
IL-1β (mus)	TTCAGGCAGGCAGTATCACTC	GAAGGTCCACGGGAAAGACAC
CCL20 (mus)	GCCTCTCGTACATACAGACGC	CCAGTTCTGCTTTGGATCAGC
CCR6 (mus)	CCTGGGCAACATTATGGTGGT	CAGAACGGTAGGGTGAGGACA
TLR2 (mus)	GCAAACGCTGTTCTGCTCAG	AGGCGTCTCCCTCTATTGTATT
TLR7 (mus)	ATGTGGACACGGAAGAGACAA	GGTAAGGGTAAGATTGGTGGTG
TLR9 (mus)	ATGGTTCTCCGTCGAAGGACT	GAGGCTTCAGCTCACAGGG
GAPDH (mus)	CTCCCACTCTTCCACCTTCG	GGCCTCTCTTGCTCAGTGTC

### Western Blotting

Stomach tissues were immersed in NP40 lysis buffer (Solarbio, China) supplemented with protease and phosphatase inhibitors, and sonicated on ice. The homogenates were centrifuged at 15,000× *g* for 15 min, and the supernatants were aspirated. The protein concentration was assessed using bicinchoninic acid protein assay kit (Beyotime, China), and equal amounts of protein per sample were fractionated by sodium dodecyl sulfate polyacrylamide gel electrophoresis using a 10% gel. The protein bands were transferred onto nitrocellulose filter membranes, blocked with 5% non-fat milk for 1 h at room temperature, and then incubated overnight with anticleaved caspase-1 (Asp296; CST, Cat. no. 67314), anticaspase-1 (Abcam, Cat. no. ab138483), anti-IL-1β (3A6; CST, Cat. no. 12242), anticleaved-IL-1β (Asp117; CST, Cat. no. 52718), antinuclear factor kappa B (anti-NF-κB) p65 (Abcam, Cat. no. ab32536), and anti-NLRP3 (Abcam, Cat. no. ab210491) primary antibodies at 4°C. The membranes were then probed with horseradish peroxidase-conjugated secondary antibodies for 1 h at room temperature and visualized using chemiluminescent reagents (Millipore, USA). β-Actin was used as the internal control.

### ELISA

The gastric homogenates were processed as above, and the levels of IL-17, IL-23, and IL-1β were analyzed using commercially available sandwich ELISA kits (eBioscience, USA) in accordance with the manufacturer's instructions. Absorbance was measured at 450 nm using Synergy H1 Hybrid Microplate Reader (BioTek, USA), and the cytokine concentrations were calculated from a standard curve.

### Statistical Analysis

Data were expressed as mean ± standard deviation (SD) and evaluated by the Fisher's exact test, Student's *t*-test, and Mann-Whitney test as appropriate. All tests were performed using GraphPad Prism 6.0 system. Two-tailed *P* < 0.05 were considered statistically significant.

## Results

### MDSCs Infiltrate in Human MALT Lymphoma Tissues

**Figure 1:**
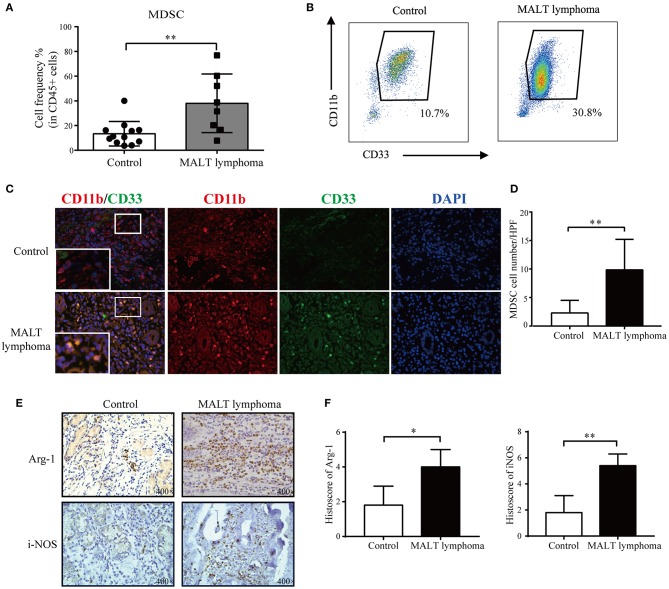
The expansion and activation of MDSCs in patients with MALT lymphoma. **(A)** The proportion of myeloid-derived suppressor cells (MDSCs) in CD45^+^ peripheral blood mononuclear cells (PBMCs) is higher in newly diagnosed mucosa-associated lymphoid tissue (MALT) lymphoma patients (*n* = 8) compared to age-matched controls (*n* = 12); **P < 0.01. **(B)** Representative flow cytometric dot plots showing MDSCs in the peripheral blood of a patient and age-matched healthy individual. **(C)** Representative immunofluorescence images of CD11b^+^CD33^+^ MDSCs in gastric MALT lymphoma and paired normal tissues. Red, CD11b; green, CD33; and blue, 4′ ,6-diamidino-2-phenylindole (DAPI); original magnification, 400×. **(D)** Quantitative analysis of MDSCs (CD11b^+^CD33^+^) in gastric MALT lymphoma and paired normal tissues; **P < 0.01. **(E)** Representative immunohistochemistry (IHC) images of arginase 1 (Arg-1) and inducible nitric oxide synthase (iNOS) in gastric MALT lymphoma and paired normal tissues. Original magnification, 400×. **(F)** Histoscores of Arg-1 and iNOS were higher in gastric MALT lymphoma tissues compared to control; **P < 0.01, *P < 0.05.

The proportion of circulating MDSCs (CD45^+^lin^−^CD33^+^HLA^−^DR^−^CD11b^+^) was significantly higher in the MALT lymphoma patients compared to that in healthy donors (Figures 1A,B). The gating strategy of MDSCs is shown in Figure S1A. Furthermore, the CD33^+^CD11b^+^ MDSCs were also enriched in the gastric MALT lymphoma biopsies (Figures 1C,D), which correlated with significant upregulation of Arg-1 and iNOS compared to paired normal gastric tissues (Figures 1E,F). Since Arg-1/iNOS overexpression is a key mechanism through which MDSCs mediate local immune suppression (9–11), it is likely involved in creating an immunosuppressive MALT lymphoma microenvironment as well. In addition, the proportion of circulating Tregs was also significantly elevated in the MALT lymphoma patients compared to healthy donors (Figure S1B). Taken together, MDSCs are enriched in MALT lymphoma and are responsible for the immunosuppressive conditions.

### Persistent *H. felis* Infection Induces Lymphoepithelial Lesions in Murine Stomach

**Figure 2:**
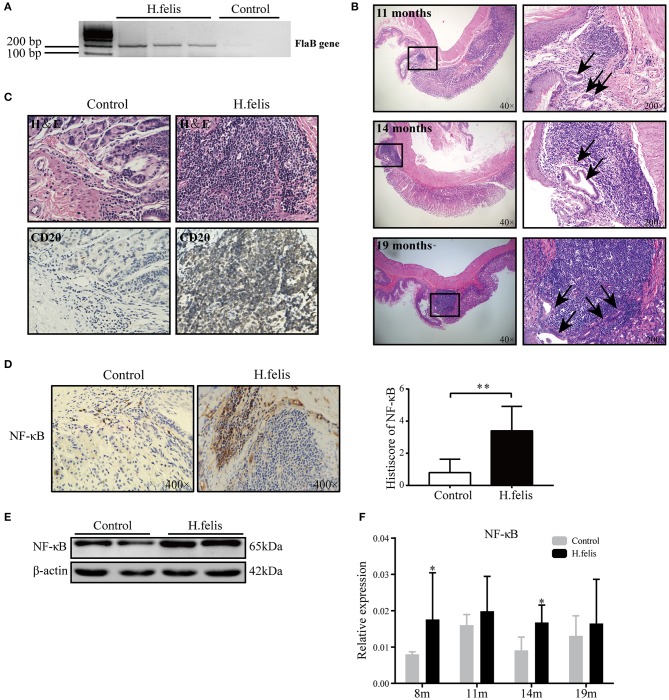
*H. felis* infection upregulated nuclear factor kappa B (NF-κB) signaling and induced lymphoid aggregates. **(A)** *FlaB* messenger RNA (mRNA) expression in the stomach of *H. felis*-infected mice 1 month postinfection. **(B)** Representative H&E-stained images of infected stomach showing lymphoid follicles with expanded marginal zone and infiltrating centrocyte-like cells (arrows) at 11 months postinfection. Lymphoepithelial lesions (LELs) (arrows) and infiltrating lymphocytes were seen in the gastric glands at 14 and 19 months postinfection. Original magnification, 400×. **(C)** Representative immunohistochemistry (IHC) images showing CD20^+^ B cells in lymphoid aggregates. **(D)** Representative IHC images (left) showing NF-κB expression in the stomach of *H. felis*-infected and control mice and bar diagram (right) summarizing the histoscores in both groups; **P < 0.01. **(E)** Immunoblot showing gastric NF-κB protein levels in *H. felis*-infected and control mice. **(F)** The NF-κB mRNA levels at 8, 11, 14, and 19 months postinfection; *P < 0.05.

To determine the possible pathogenic role of MDSCs in MALT lymphomagenesis, we infected mice with *H. felis* to mimic gastric MALT lymphoma development. The presence of *Helicobacter* strain-specific gene *FlaB* in the gastric tissues of these mice indicated successful infection (Figure 2A). Furthermore, the urease test showed that the infection rates of *H. felis* were, respectively, 75, 100, 90, and 100% at 8, 11, 14, and 19 months postinfection (Figure S2A). Furthermore, the infected mice developed lymphoid follicles with classical features like germinal center and mantle zone from 8 months after bacterial gavage, whereas no pathological changes were detected in the uninfected mice (Figure S2B). The pathological damage gradually increased in a time-dependent manner, and after 11 months of infection, the marginal zone expanded with infiltrating centrocyte-like cells that destroyed the gastric glands. LELs, a characteristic feature of MALT lymphoma, were observed 14 months postinfection (Figure 2B). These changes simulated the histopathological characteristics of MALT lymphomagenesis. Furthermore, the lymphoid aggregates consisted predominantly of B cells (Figure 2C), along with some T cells (CD3^+^) and very few macrophages (F4/80^+^) (Figure S2C). The NF-κB pathway, a critical mediator of the inflammatory response, was also activated in the infected mice (Figures 2D–F), indicating an important role in MALT lymphomagenesis.

### MDSCs Are Enriched in the Gastric LELs of *H. felis*-Infected Mice

**Figure 3:**
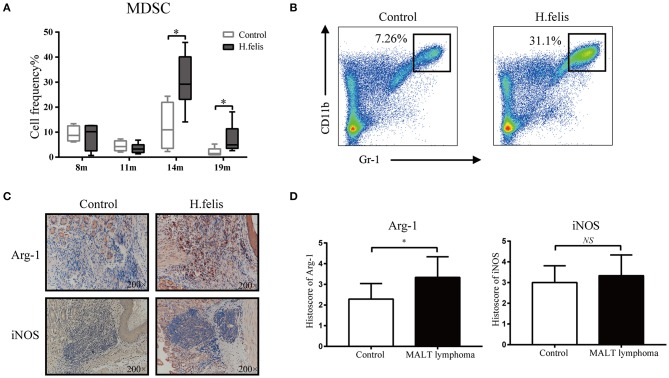
*H. felis* infection induced myeloid-derived suppressor cells (MDSCs) expansion and upregulated arginase 1 (Arg-1) and inducible nitric oxide synthase (iNOS). **(A)** Percentage of MDSCs in the peripheral blood of control and *H. felis*-infected mice at 8 months (control, *n* = 4; *H. felis, n* = 4), 11 months (control, *n* = 4; *H. felis, n* = 6), 14 months (control, *n* = 4; *H. felis, n* = 9), and 19 months (control, *n* = 6; *H. felis, n* = 8) postinfection; *P < 0.05. **(B)** Representative flow cytometry dot plots showing MDSCs in the *H. felis*-infected and control mice at 14 months postinfection. **(C)** Representative IHC images showing Arg-1 and iNOS expression in the stomach of *H. felis*-infected and control mice. Original magnification, 200×. **(D)** Comparison of Arg-1 and iNOS levels in both groups, *P < 0.05.

To determine whether the histological alterations in *H. felis*-infected mice were also associated with MDSCs enrichment as observed in the MALT lymphoma tissues, we analyzed the distribution and percentage of MDSCs at various time points postinfection. At the early-stage of infection (8–11 months), only mild lesions were seen in the stomach, and the number of MDSCs in peripheral blood was unaffected. However, at the later stages (14–19 months), the circulating MDSCs increased significantly and was accompanied by gastric accumulation of Gr-1^+^CD11b^+^ MDSCs (Figures S3A,B) and severe LELs (Figures 3A,B). Furthermore, Arg-1 was significantly upregulated in the stomach of *H. felis*-infected mice, indicating MDSCs activation in response to gastric MALT lymphoma development (Figures 3C,D), whereas iNOS levels were not altered. This suggested that Arg-1 was the primary immunosuppressive factor employed by MDSCs.

### The γδT17 Cells Are Enriched in *H. felis*-Infected Mice

**Figure 4:**
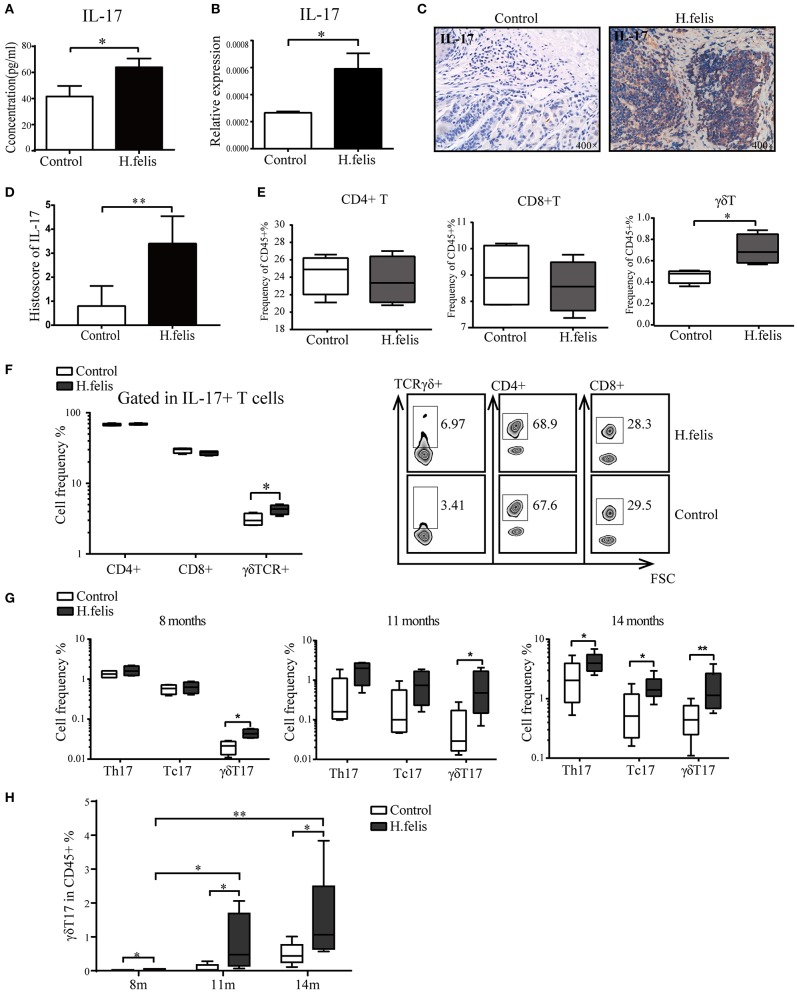
γδT17 cells were elevated in *H. felis*-infected mice. **(A)** Interleukin-17 (IL-17) levels in the gastric homogenates at 8 months postinfection. **(B)** IL-17 messenger RNA (mRNA) level in the stomach at 8 months postinfection. **(C)** Representative immunohistochemistry (IHC) image showing *in situ* IL-17 in the stomach of *H. felis*-infected and control mice and **(D)** corresponding histoscores. Original magnification, 400×. **(E)** Percentage of CD4^+^, CD8^+^, and γδT cells among CD45^+^ cells in the spleen of *H. felis*-infected mice and control mice at 8 months postinfection. **(F)** Percentage of CD4^+^IL-17^+^(Th17), CD8^+^IL-17^+^(Tc17), and TCRγδ^+^IL-17^+^(γδT17) cells in the spleen from *H. felis*-infected and control mice at 8 months (left), and representative flow cytometric plots of the same gated with IL-17^+^CD3^+^ T cells (right). **(G)** The percentage of Th17, Tc17, and γδT17 cells among CD45^+^ cells in the spleen at 8, 11, and 14 months postinfection. **(H)** Time-dependent increase in γδT17 cells with the progression of chronic *H. felis* infection; 8 months (control, *n* = 4; *H. felis, n* = 4), 11 months (control, *n* = 5; *H. felis, n* = 4), and 14 months (control, *n* = 5; *H. felis, n* = 10). *P < 0.05, **P < 0.01.

IL-17, an important proinflammatory cytokine, exerts crucial functions in carcinogenesis and tumor growth (18) by recruiting MDSCs at tumor sites (14, 15, 19). We detected a significant upregulation in IL-17 protein and messenger RNA (mRNA) levels in the gastric homogenates of infected mice at 8 months postinfection (Figures 4A,B). Furthermore, IL-17 was enriched in the gastric lymphoid aggregates of the infected mice (Figures 4C,D). Since IL-17 could be secreted by several cell types, including CD4^+^ T cells (Th17), CD8^+^ T cells (Tc17), and γδT cells (γδT17), we assessed the changes of CD4^+^, CD8^+^, and γδT cells following *H. felis* infection. The results showed a significant increase in γδT cells population, while the percentages of CD4^+^ and CD8^+^ T cells were similar to that in the control group (Figure 4E). We next compared the proportion of CD4^+^, CD8^+^, and γδT cells among the splenic IL-17-producing T cells and found that, although the Th17 cells were the predominant type, the difference was not significant between the *H. felis*-infected and control mice. However, the number of splenic γδT17 cells increased significantly after *H. felis* infection (Figure 4F) and is possibly the main immune regulatory cell population in *H. felis*-induced pathologies. We subsequently compared the proportion of splenic Th17, Tc17, and γδT17 cells at different time points and detected increased numbers of γδT17 cells at 8, 11, and 14 months postinfection. No differences were seen in the Th17 and Tc17 populations between the *H. felis*-infected and control mice except at 14 months postinfection (Figure 4G). The proportion of splenic γδT17 cells increased in a time-dependent manner during chronic *H. felis* infection (Figure 4H), indicating a possible relationship between systemic γδT17 cells and the development of gastric MALT lymphoma.

### IL-1β Is Activated in Gastric Mucosa After Long-Term *H. felis* Infection

**Figure 5:**
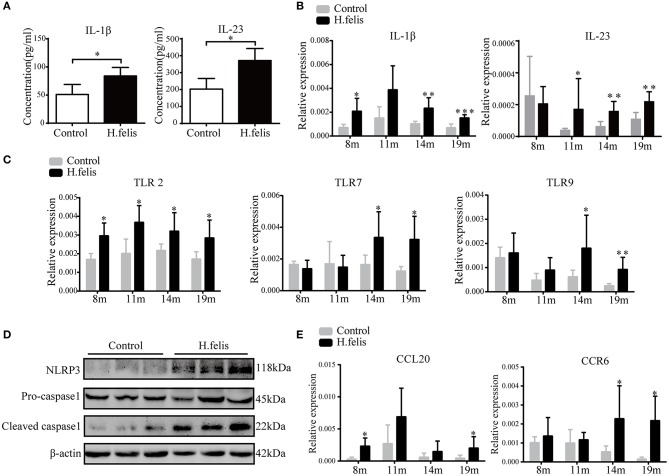
*H. felis*-infected mice show interleukin (IL)-1β activation and chemokine stimulation. **(A)** IL-1β and IL-23 levels in the gastric homogenates at 8 months postinfection. **(B)** IL-1β and IL-23 messenger RNA (mRNA) levels in the gastric tissue at 8, 11, 14, and 19 months postinfection. **(C)** TLR2, TLR7, and TLR9 mRNA levels at various time points after infection. **(D)** Immunoblot showing NLRP3 inflammasome expression in the stomach of *H. felis*-infected and control mice. **(E)** CCL20 and CCR6 mRNA levels in both groups. Data are shown as mean ± SD, *P < 0.05, **P < 0.01, ***P < 0.001.

IL-1β and IL-23 stimulate γδT cells to secrete IL-17 in multiple experimental models (15, 20). We found that both were significantly elevated in the gastric homogenates of *H. felis*-infected mice at 8 months postinfection (Figures 5A,B). Studies show that the cytokine response to *Helicobacter* infections is mediated by Toll-like receptor 2 (TLR2) (21). Consistent with this, TLR2, TLR7, and TLR9 were upregulated in the gastric mucosa after *H. felis* infection (Figure 5C). In respiratory bacterial infections, NLRP3 inflammasome-dependent IL-1β regulates the γδT17 cell response (22). In the mice with persistent *H. felis* infection as well, we observed a marked increase in the levels of caspase-1 and NLRP3 (Figure 5D), indicating activation of the NLRP3 inflammasome and subsequent IL-1β activation. Since the γδT17 cells are CD27^−^CCR6^+^ (23) and are recruited by the CCL20-CCR6 axis (24, 25), we also evaluated the expression levels of these chemokines in the gastric mucosa. As shown in Figure 5E, CCR6 and CCL20 levels were significantly higher in the *H. felis-*infected gastric tissue at 19 months postinfection, suggesting that γδT17 is recruited to the gastric tissues by these chemokines.

### The γδT17 Cells Infiltrate Into Human MALT Lymphoma Tissues

**Figure 6:**
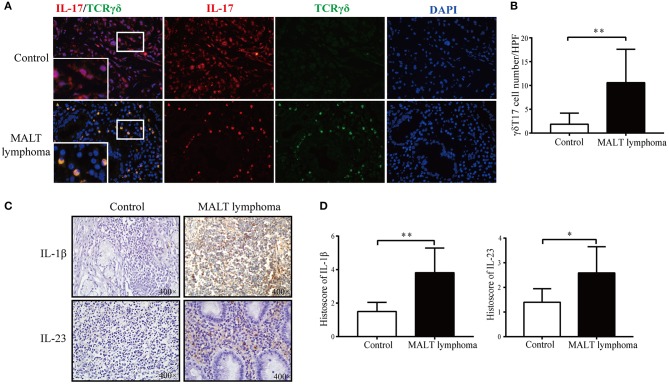
The expansion of γδT17 cells and upregulation of interleukin (IL)-1β and IL-23 in mucosa-associated lymphoid tissue (MALT) lymphoma patients. **(A)** Representative immunofluorescence images showing γδT17 (TCRγδ^+^IL-17^+^) cells in gastric MALT lymphoma tissues and paired normal tissue. IL-17 in red, TCRγδ in green, and 4′ ,6-diamidino-2-phenylindole (DAPI) in blue; original magnification, 400×. **(B)** Percentage of γδT17 (TCRγδ^+^IL-17^+^) cells in the above groups. **(C)** Representative immunohistochemistry (IHC) images showing *in situ* IL-1β and IL-23 in gastric MALT lymphoma tissues and paired normal gastric tissues. **(D)** Histoscores of IL-1β and IL-23 were statistically higher in gastric MALT lymphoma tissues than control. Original magnification, 400×; **P < 0.01, *P < 0.05.

Consistent with the observations in the murine model, there was an obvious infiltration of γδT17 cells in the MALT lymphoma tissues (Figures 6A,B). Furthermore, IL-1β and IL-23 levels were also higher in the MALT lymphoma compared to the paired normal tissues (Figures 6C,D). However, there was no significant difference in the number of peripheral Th17, Tc17, and γδT17 cells between the patients and controls (Figure S4), suggesting that the tumor-infiltrating rather than peripheral γδT17 cells regulate the development of MALT lymphoma.

## Discussion

Chronic inflammation is the pathological basis of multiple solid tumors, wherein immune dysfunction triggers malignant transformation of cells. Mounting evidence indicates that MDSCs are enriched both locally and systemically during cancer growth in mice and humans (7, 9–12, 26), and their activation in pathological conditions upregulates Arg-1 and iNOS. Arg-1 consumes l-arginine, an intermediate for TCR biosynthesis, resulting in failure of T-cell proliferation and activation (26). Furthermore, the nitric oxide produced by iNOS induces CD8^+^ T-cell apoptosis by downregulating CD44 and CD62L, upregulating CD95, and inhibiting JAK3 and STAT5 signaling (27). In an A20 lymphoma murine model, MDSCs induced an immunosuppressive microenvironment through the activation of Tregs (10). In the present study as well, MDSCs accumulated in large numbers in MALT lymphoma tissues and expressed high levels of Arg-1 and iNOS, suggesting an important role in the development of MALT lymphoma.

Gastric *H. felis* infection simulates the pathological changes associated with chronic gastritis, lymphoid follicle formation, and LELs during gastric MALT lymphoma development (16, 28–30). The histopathological changes in our murine model were consistent with previous studies (16, 28). Although the stomach is *a priori* devoid of lymphoid tissue, we observed lymphoid hyperplasia in the chronically infected mice. Lymphoid follicles appeared from 8 months postinfection, and the gastric glands were invaded by multiple lymphocytes 14 months after infection. These LELs are an important pathological feature of gastric MALT lymphoma and predominantly consisted of B cells. The lymphoid aggregates in the infected mice were also enriched with MDSCs expressing high levels of Arg-1 and iNOS. Interestingly, the significant expansion of MDSCs was observed from 14 months postinfection and coincided with severe tissue damage and LELs, suggesting a possible relationship between MDSCs increment and severity of disease.

The number of γδT17 cells also increased significantly in the gastric lesions of the *H. felis*-infected mice, as well as in clinical gastric MALT lymphoma specimens. Although γδT cells and IL-17 are potent antitumor effectors (31), studies also report protumor effects of the γδT17 cells (14, 15). In animal models of fibrosarcoma, skin cancer, and ovarian cancer, γδT17 cells infiltrate the tumors and secrete IL-17, thereby promoting tumor growth (32, 33). Furthermore, recent studies show that γδT17 mediate immune dysfunction in the tumor microenvironment by recruiting MDSCs (14, 15, 34). Furthermore, IL-23 induces γδT17 cells to secrete IL-17, IL-8, tumor necrosis factor α, and granulocyte-macrophage colony-stimulating factor, consequently recruiting and activating MDSCs in colorectal tumors (14). In addition, activated γδT17 cells in the liver tumor microenvironment produced large amounts of IL-17 and recruited MDSCs, which enabled tumor cells to escape the immune surveillance by inhibiting CD8^+^ T cell functions (15). In the present study, we found that the proportion of γδT17 increased as *H. felis* infection progressed, indicating a possible relationship between these cells and the pathological gastric lesions. As observed with the MDSCs, γδT17 numbers markedly increased with the deterioration of lymphoepithelial defects. In view of these observations, we hypothesized that γδT17 cells were involved in the malignant transformation of the inflamed gastric epithelium during persistent *Helicobacter* infection. However, the evidence is insufficient at present to conclude that γδT17 cells mediate immune dysregulation in MALT lymphoma by recruiting MDSCs, and further studies are warranted to validate this hypothesis.

Previous studies have shown that *Helicobacter* infection activates the innate immune system in a TLR-dependent manner, leading to activation of the NF-κB pathway and cytokine production (21). Consistent with this, we observed a significant increase in the levels of TLR2, TLR7, and TLR9 in the *H. felis*-infected gastric mucosa, along with NF-κB upregulation. The chronic inflammation and numerous genetic aberrations seen in gastric MALT lymphoma are linked with dysregulated NF-κB signaling (35). The activation of the NF-κB pathway, along with the pathological role of *Helicobacter*, indicate that chronic inflammation is essential for lymphomagenesis (5). In addition, the critical role of NF-κB in MDSCs accumulation and function has become apparent in recent years. Studies show that IL-1β activates MDSCs through the NF-κB pathway, and blocking IL-1 receptor signaling inhibited gastric preneoplasia and MDSC mobilization (36). Thus, NF-κB pathway likely regulates the immune responses in gastric MALT lymphoma through various mechanisms.

IL-1β and IL-23 are known to drive γδT17 responses and induce IL-17 production (20). In addition, MDSCs also polarize the γδT cells to the γδT17 phenotype by secreting IL-23 and IL-1β (15). Both cytokines were elevated in the *H. felis*-infected gastric mucosa, indicating γδT17 activation. In addition, NLRP3 inflammasome increases IL-18 and IL-1β production, thereby promoting tumor cell proliferation and inhibiting apoptosis during lymphoma development (37). The NLRP3 inflammasome was activated in the gastric mucosa 8 months postinfection. Based on these observations, we hypothesize that TLR2-mediated recognition of *H. felis* activates the NLRP3 inflammasome and triggers IL-1β production. Chemokines such as CCL20 then recruit γδT17 cells to the gastric lesions and aggravate immunosuppression.

In conclusion, we provided evidence of the enrichment of γδT17 and MDSCs in *Helicobacter*-induced MALT lymphomagenesis. Our findings highlight the therapeutic potential of modulating γδT17 cells and MDSCs in gastric MALT lymphoma.

## Data Availability Statement

The datasets generated for this study are available on request to the corresponding author.

## Ethics Statement

The studies involving human participants were reviewed and approved by Medical Ethical Committee of Qilu Hospital, Shandong University. The patients/participants provided their written informed consent to participate in this study. The animal study was reviewed and approved by Medical Ethical Committee of Qilu Hospital, Shandong University.

## Author Contributions

CJ, YZ, and MJ designed the research and analyzed the data. YZ, FL, YP, and LW performed the experiments. JY, GL, JL, TS, and DM provided scientific suggestions and supervised the project. YZ wrote the manuscript. CJ, FL, MJ, and YW contributed to the manuscript revision. All authors critically reviewed the article and approved the final manuscript.

### Conflict of Interest

The authors declare that the research was conducted in the absence of any commercial or financial relationships that could be construed as a potential conflict of interest.
