# Supplementary material for: Myeloid-Derived Suppressor Cells and γδT17 Cells Contribute to the Development of Gastric MALT Lymphoma in H. felis-Infected Mice
Source: Front Immunol. 2020 Jan 28;10:3104. doi: 10.3389/fimmu.2019.03104 (PMC6998799; doi:10.3389/fimmu.2019.03104)
Supplement: Supplementary file 1 [file Data_Sheet_1.pdf]

Supplementary Material

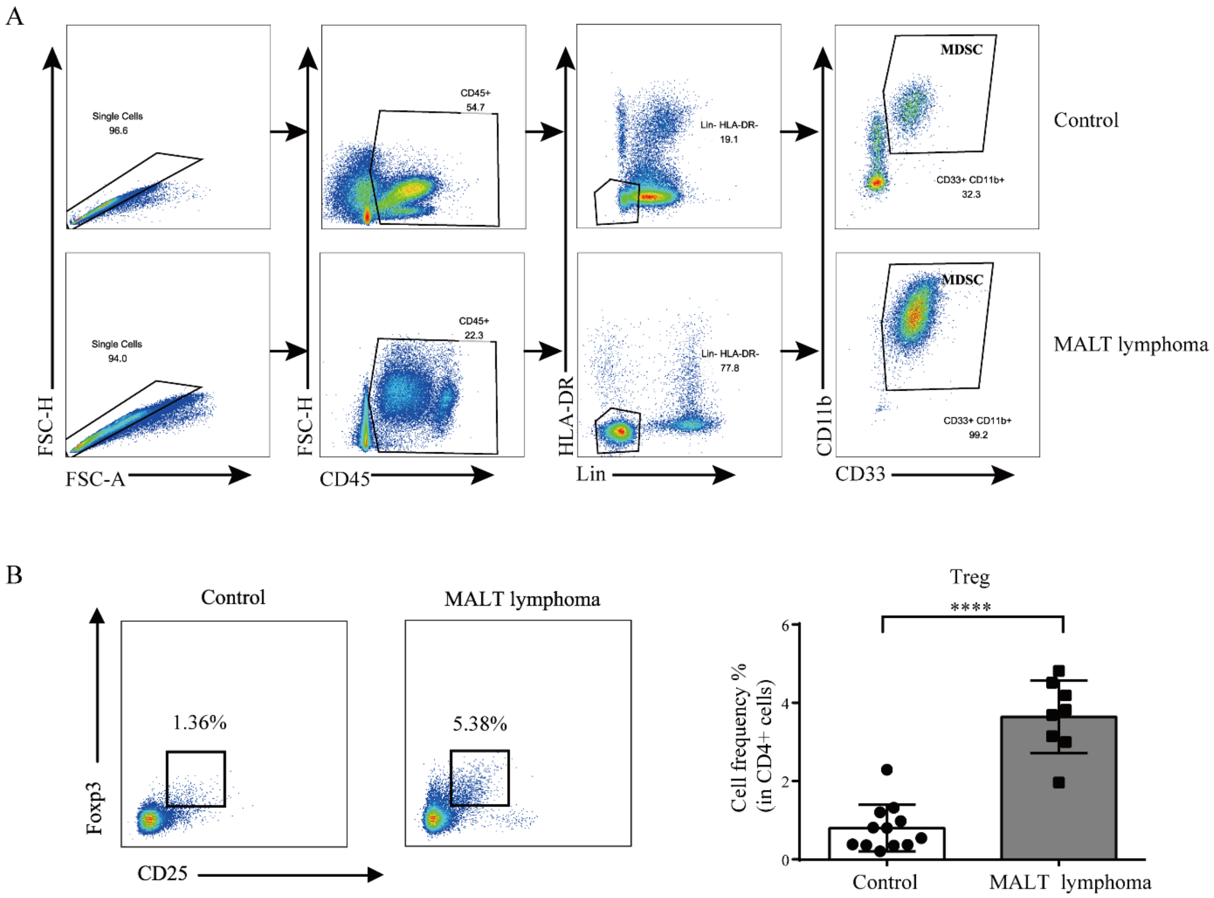

Figure S1. MDSCs and Treg are significantly increased in patients with MALT lymphoma. Related to Figure 1.

(A) MDSCs gating strategy and representative flow cytometric analysis of MDSCs in a MALT lymphoma patient and a healthy individual. (B) Tregs were assessed by flow cytometry in peripheral blood of patients and healthy individuals. Left panel shows representative flow cytometric analysis. Right panel shows bar diagram that summarizes the percentages of $CD25^{+}Foxp3^{+}$ Treg among $CD4^{+}$ T cells from patients with MALT lymphoma (n = 8) and age-matched controls (n = 12); $^{**}P < 0.01$, $^{****}P < 0.001$.

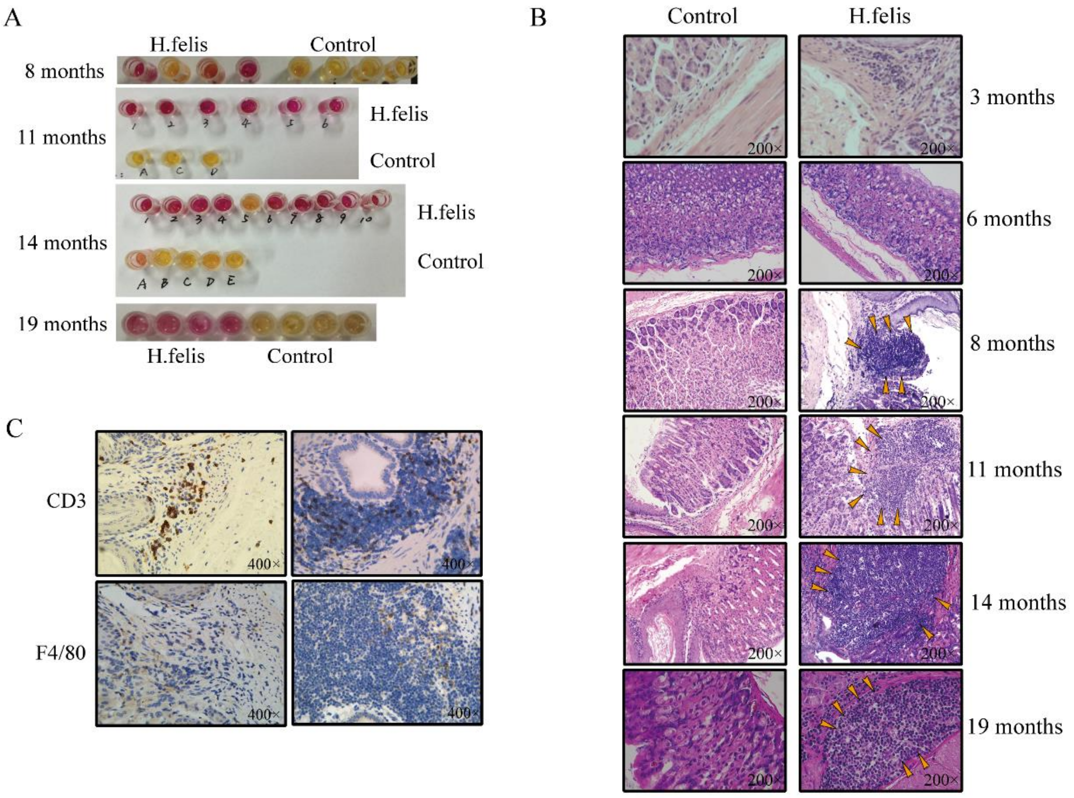

Figure S2. ***H. felis*** **colonization and histopathological changes in infected stomach.**

(A) *H. felis* colonization was verified with a rapid urease test at various time points. Results were considered as positive following the appearance of definite red color. (B) Histopathological changes in the gastric mucosa of *H. felis*-infected and control mice at various time points. Orange arrows indicate the formation of lymphoid follicles or lymphoepithelial lesions. H&E staining; original magnification, 200× (stomach). (C) Representative Immunohistochemical assessment of CD3 and F4/80 expression in the position of lymphoid aggregates.

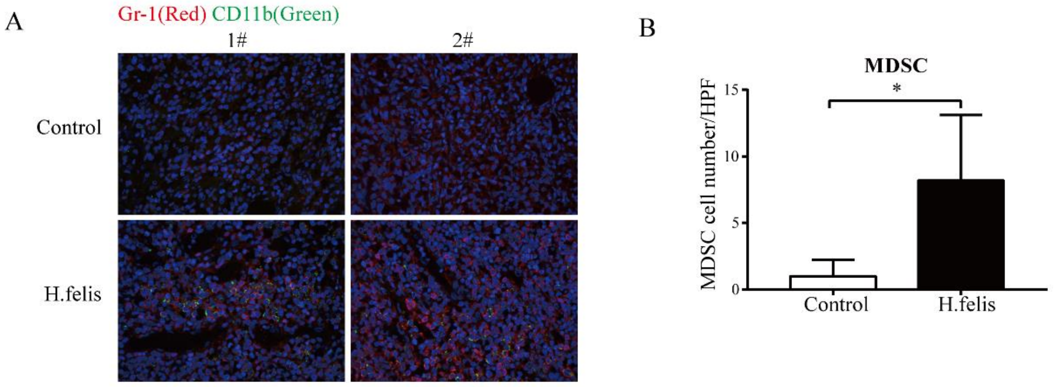

Figure S3. ***H. felis*** **infection induced MDSCs expansion in stomach.**

(A) Representative immunofluorescence microscopy images of MDSCs (Gr-1^{+}CD11b^{+}) in stomach of *H. felis*-infected and control mice. Gr-1 in red, CD11b in green and DAPI in blue; Original magnification, 400×. (B) Quantitative analysis of MDSCs (Gr-1^{+}CD11b^{+}) in stomach of *H. felis* infected and control mice; $^{*}P < 0.05$.

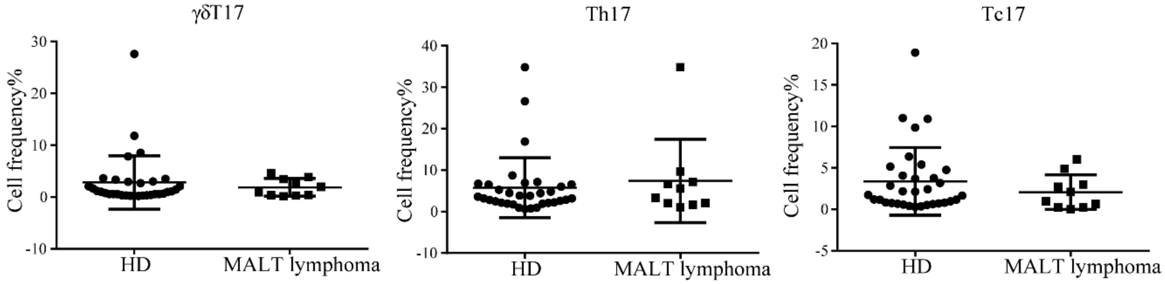

Figure S4. The percentage of Th17, Tc17 and γδT17 in CD45^{+} PBMCs were detected by flow cytometry, with no significance among MALT lymphoma patients and healthy individuals.
